# Reproductive health interventions for Inuit youth in the north: a scoping review

**DOI:** 10.1186/s12978-021-01119-6

**Published:** 2021-03-20

**Authors:** Hannah Mikhail, Sarah E. Kelly, Colleen M. Davison

**Affiliations:** 1 Department of Public Health Sciences, Queen’s University, 62 Fifth Field Company Lane, Carruthers Hall #203, Kingston, ON K7L3N6 Canada; 2 Department of Global Development Studies, Queen’s University, Kingston, ON Canada

**Keywords:** Adolescent health, Indigenous health, Inuit health, Northern health, Reproductive health

## Abstract

**Background:**

Inuit have thrived in the northern regions of Canada and Alaska for thousands of years. Recent evidence suggests that Inuit in this region have experienced systemic barriers to reproductive health with resulting disparities in reproductive health-related outcomes including those among youth. Northern youth-focused reproductive health intervention research or evaluations have not to date been well summarized. The objective of this scoping review was to summarize the literature over the past twenty years focusing on reproductive health interventions for adolescents in northern Inuit communities.

**Methods:**

English-language articles from 2000 to 2020 were identified from seven scientific databases, a general internet search and a review of relevant websites. Two reviewers screened titles, abstracts and full texts and included articles if they mentioned a reproductive health intervention and pertained, directly or indirectly, to reproductive health for Inuit aged 10–19 in northern communities.

**Results:**

Seventeen articles met the inclusion criteria, across six themes: (1) Barriers to reproductive health interventions in the north; (2) Northern midwifery; (3) Northern birthing centres; (4) Fetal fibronectin tests for identifying high-risk pregnancies; (5) Prenatal education classes; and (6) Interventions to improve access to and quality of reproductive health supports*.*

**Conclusion:**

Overall there is relatively limited evidence base specific to reproductive health interventions and northern Inuit youth. What does exist largely focuses on maternal health interventions and is inclusive of but not specific to youth. There is some evidence that youth specific educational programs, participatory action research approaches and the promotion of northern birthing centres and midwifery can improve reproductive health for adolescents and young mothers in northern Inuit communities. Future initiatives should focus on the creation and evaluation of culturally relevant and youth specific interventions and increasing community and youth participation in intervention research for better reproductive health.

## Plain English summary

Inuit living in northern regions of Canada and Alaska have succeeded for thousands of years and have showed much resilience. Despite this, there is evidence of differences in reproductive health outcomes between Inuit and non-Inuit living both in the north as well as in more southern regions of North America. Research shows that northern Inuit unfortunately have poorer reproductive health outcomes and have more limited access to health programs and services. This scoping review summarized available evidence from the past twenty years focusing specifically on the reproductive health interventions for adolescents in Inuit communities in North America. The articles focused on specific interventions as well as barriers to the success of reproductive health interventions. This scoping review found that there is limited evidence overall about reproductive health interventions for northern Inuit youth. The existing interventions largely focus on maternal health topics and have a general, non-youth specific, focus. Involving Inuit youth in future intervention research, creating culturally-appropriate and youth-specific reproductive health educational resources, and promoting northern birthing centres and midwifery services could improve reproductive health for Inuit youth.

## Introduction

Despite a deeply troubling history of colonization, forced settlement, relocation and residential schools, Inuit in northern regions of North America have shown great resilience. Unfortunately, structural barriers to healthcare and disparities in the social determinants of health have meant that Inuit youth and young mothers are at a much higher risk of adverse health outcomes as compared to their non-Indigenous counterparts and those in southern regions [1]. Canadian Inuit women in the north experience higher rates of pregnancy as teenagers compared to national rates [2]. Historically, Inuit communities in North America have faced inequities in terms of access to healthcare and health services, including those pertaining to reproductive health [3]. Most Inuit women living in remote northern communities are relocated to an urban centre to give birth [3]. In the first half of the 1900s, this pattern was different, with the majority of northern Inuit women giving birth in their communities with the help of midwives or other females. In the 1970s, it was deemed unsafe for women in the remote north to give birth in facilities that were not supplied with specific types of obstetrical equipment [1, 4]. Governments also reasoned that northern community health centres and midwives were not able to properly care for mothers with high-risk pregnancies or premature infants [5]. Since then, in an effort to provide “universal care”, most Inuit expectant mothers living in northern communities are transferred to urban health centres at around 36 weeks’ gestation in order to give birth [6].

Reproductive health is defined by the World Health Organization as a state of physical, mental, and social well-being and not merely the absence of disease or infirmity, in all matters relating to the reproductive system and to its functions and processes [7]. Reproductive health encompasses a number of service areas including maternal, child and newborn health, family planning, pre- and post-natal care, gender-based violence, prevention and management of abortion, fertility concerns and reproductive education. This scoping review aimed to summarize published literature from the past 20 years that focused on reproductive health interventions for Inuit youth living in northern communities. Interventions were broadly defined and could include any intentional service, program or activity relevant to any aspect of reproductive health. The review was designed in response to needs expressed by northern community service providers and the hope is that it provides a summary of recent intervention studies, helps identify key gaps in the literature, and could be useful for informing future interventions, services, research, and resource allocations for northern Inuit youth.

## Methods

A scoping review that sought literature from academic databases, a general internet search and a targeted search of relevant websites was undertaken [8]. English-language articles published between 2000 and 2020 that pertained to Inuit youth (aged 10–19) in northern communities in North America were included. As sexual and reproductive health are often combined, articles that pertained to either or both of these topics areas were initially included [9]. For final inclusion, articles had to include mention of a specific reproductive health program or intervention. Peer-reviewed publications fitting these criteria were identified using Embase (Ovid), Medline (Ovid), ERIC (EBSCO), CINAHL (EBSCO), PsycINFO, Web of Science, ProQuest, Google Scholar and PubMed. The full Embase search strategy with keywords is provided in Table 1.

**Table 1:** Embase Search Strategy

inuk* OR inuit* OR (north* adj5 indigenous) OR eskimo* OR inupiat* OR aleut* OR kalaallit* OR “exp eskimo-aleut people”	**AND**	“sexuality/ or exp sexual behaviour/ or sexual health/ or exp sexual orientation/ or transsexuality” OR “exp sexually transmitted disease” OR “reproductive health” OR “exp pregnancy” OR “exp birth control” OR “exp sexual assault” OR “sexual education” OR sex* OR gender* OR two spirit* OR pregnan* OR reproduct*	**AND**	“education/ or education program/ or education model/ or exp health education/ or learning environment/ or mentoring/ or “outcome of education”/ or sexual education” OR “health service/ or family service/ or health services research/ or maternal health service/ or public health service/ or school health service” OR “health education/ or breastfeeding education/ or childbirth education/ or health literacy/ or hiv education/ or parenting education/ or patient education/ or school health education” OR “public health campaign/ or social marketing/ or public health message” OR “teaching” OR educat* OR teach* OR learn* OR inform* OR resourc* OR tool* OR program* OR lesson*

Comparable searches were performed in the remaining databases. A search was also conducted using the websites of relevant organizations (Open Grey, Centre for Northern and Rural Health Research, Institute for Circumpolar Health, and Arctic Institute of Community Based Research), the reference lists of the identified articles and a general Internet search [10]. Papers that focused on: (1) sexual health only, (2) adults only; (3) children under 10 only; (4) non-Indigenous individuals only; (5) not a northern, rural, remote or isolated community; (6) had missing information or not focused on a program or intervention related to reproductive health; (7) non-English articles; and (8) articles that were published prior to the year 2000, were excluded. Articles where full text could not be accessed either online or through an academic librarian were also excluded. Articles were individually screened by two reviewers (Authors #1 and #2), using the software platform Covidence [11]. Author #3 assisted with reconciling any inclusion differences between the initial two screeners. After consensus on abstract and title screening was reached, the full texts of the remaining articles were reviewed for inclusion and exclusion criteria and data was extracted and summarized from all the included studies.

## Results

**Fig. 1:**
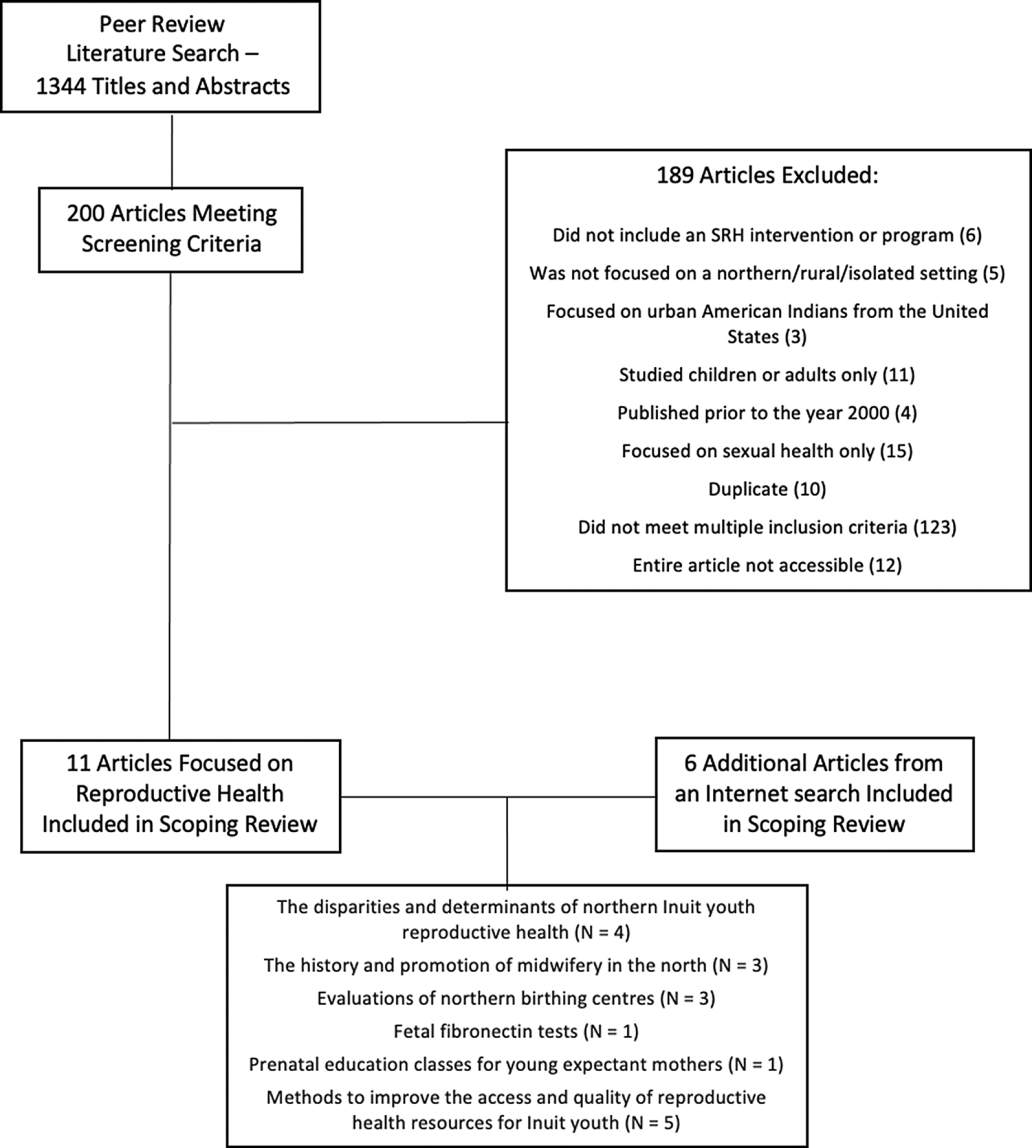
Screening results

A total of 1344 articles were initially discovered during the scoping review. A total of 200 articles were reviewed in full text for inclusion/exclusion. Eleven articles were found in the scientific database search while an additional six articles were found in the literature search beyond the initial scientific databases. This resulted in a total of 17 articles meeting the inclusion criteria overall. The flowchart of the screening process for this review are illustrated in Fig. 1.

The 17 articles in this reproductive health review were separated into six themes. These themes were separated based on the article’s main focus and were not necessarily mutually exclusive. These themes included: (1) Barriers to reproductive health interventions in the north; (N = 4); 2. Northern midwifery (N = 3); 3. Northern birthing centres (N = 3); 4. Fetal fibronectin tests for identifying high-risk pregnancies (N = 1); 5. Prenatal education classes (N = 1); and 6. Methods to improve the access to and quality of reproductive health supports (N = 5). The articles in this scoping review varied in study design, ranging from cross-sectional (N = 13), retrospective (N = 2), analytic historical study (N = 1), case study (N = 1). Many of the articles (N = 11) in this review did not specify age, but studied expectant mothers of childbearing age which would include young mothers as well. Due to the fact that rates of teenage pregnancy are higher in northern Inuit communities compared to national averages [3], it can be assumed that a proportion of these expectant mothers would be youth and we determined these articles to be relevant to northern Inuit youth and they were included. Details of each of the included articles and the interventions that they described are provided in Table 2.

**Table 2:** A summary of results and interventions from the included articles

Author(s) / year	Adolescent specific?	Setting	Sample/ population	Topic of article	Focus of results	Description of intervention/ research
Cardinal (2018) [2]	No	Northern Canada	Women of child-bearing age in northern Canadian communities	Northern midwifery	Review, not empirical research study. Author promotes northern midwifery and education of midwives	Community-based and midwifery-driven primary health care, in order to improve Indigenous reproductive health in the north
Cavanaugh (2009) [3]	No	Northern Canada	Women of child-bearing age in northern Canadian communities	Fetal fibronectin tests for identifying high-risk pregnancies	The use of fetal fibronectin tests to prevent unnecessary travel to give birth	Using the fetal fibronectin test as a method of intervention to reduce the number of “low-risk” pregnancies being evacuated to the south
Chamberlain et al. (2001) [4]	No	Northern Canada	Nulliparous pregnant mothers in Iqaluit, Nunavut	Northern birthing centres	Comparing mothers’ birthing experience in Community A versus Community B	The establishment and effectiveness of northern-based birthing centres to better support Inuit maternal health
Corosky and Blystad (2016) [5]	Yes	Arviat, Nunavut, Northern Canada	Nine male youth aged 17–22, 10 female youth aged 16–22	Methods to improve access to and quality of reproductive health supports	How to make reproductive health resources effective for an Inuit youth target population	A community health research model was followed to generate data on youth experiences of reproductive health support access in the north
Couchie and Sanderson, (2007) [6]	No	Northern Canada	Pregnant Indigenous women in remote and rural northern communities	Northern midwifery	Policy review, not empirical research study. Author promotes northern midwifery	A review of current policies in northern Indigenous communities that recommend evacuation of pregnant women to southern Canada, in order to increase the opportunity for these women to give birth in their northern communities
Douglas (2011) [8]	No	Rankin Inlet, Nunavut, Northern Canada	Inuit women who gave birth at the Rankin Inlet Birthing Centre	Northern birthing centres	History of the Rankin Inlet birthing centre	Evaluation of an established birthing centre in order to strengthen future northern birthing centres and improve Inuit youth reproductive health
Hohman-Billmeier et al. (2016) [10]	Yes	Alaska, Northern USA	Inuit youth aged 14–19	Methods and interventions to improve access to and quality of reproductive health supports	Program evaluation	Teen pregnancy prevention program for Indigenous youth in Alaska
James et al. (2010) [11]	No	Northern Canada	Pregnant Inuit women in Nunavut	Northern midwifery	How to increase midwifery services and midwifery education in the north	Promoted midwifery as an intervention to prevent northern mothers from having to relocate to the south to give birth
Jessen et al. (2016) [13]	No	Northern regions of North America	Women of childbearing age in northern regions of North America	Methods and interventions to improve access to and quality of reproductive health supports	A call to increase sexual and reproductive publications in the Arctic	Promoted research on reproductive health within northern communities as an intervention to improve general youth reproductive health
Lauson et al. (2011) [15]	No	Nunavut, Northern Canada	Mothers and children in Nunavut	Methods and interventions to improve access to and quality of reproductive health supports	Importance of creating a maternal-child health information surveillance system in the north	Initiated a comprehensive maternal-child health surveillance system, in order to improve Inuit reproductive health in Nunavut
Lawford and Giles (2016) [16]	No	Kivalliq Inuit Centre, Winnipeg, Canada	Pregnant Inuit women evacuated to the Kivalliq Inuit Centre for birth	Prenatal education classes	Implementing prenatal education classes for pregnant women	Evaluated the prenatal education available for Inuit women evacuated for birth, in order to improve overall reproductive health
Leston et al. (2012) [17]	Yes	Alaska, Northern USA	A sample of Inuit and rural youth from five communities in Alaska, aged 15–24	Barriers to reproductive health interventions in the north	Understanding the themes of youth sexual and reproductive health	Focus groups were created to better comprehend the knowledge, attitudes, and beliefs of rural Inuit youth in Alaska surrounding reproductive health, to define the most appropriate methods to educate youth regarding these topics
Logie et al. (2019a) [19]	Yes	Northwest Territories, Northern Canada	Youth aged 13–18 in seventeen NWT communities	Barriers to reproductive health interventions in the north	Exploring factors related to sexual activity and condom use among teens	Identified findings surrounding sexual practices and condom use for youth in NWT, in order to tailor proper prevention strategies to promote healthy youth sexual and reproductive health
Logie et al. (2019b) [20]	Yes	Northwest Territories, Northern Canada	Youth aged 13–18 in seventeen NWT communities	Barriers to reproductive health interventions in the north	Exploring correlations around depression, food insecurity and condom use self-efficacy	Examined factors associated with condom use self-efficacy in NWT youth, in order to inform strategies to promote sexual and reproductive health for northern youth
Luo et al. (2010) [21]	No	Northern Canada	Young Inuit mothers in northern Canada	Methods and interventions to improve access to and quality of reproductive health supports	Comparing birth outcomes among Indigenous and non-Indigenous Canadians	Gathered more data on birth outcomes in Inuit populations as a knowledge intervention to promote and inform activities to improve Inuit reproductive health in northern Canada
Moisan et al. (2016) [22]	Yes	Northern regions of North America	Inuit women aged 15–19	Barriers to reproductive health interventions in the north	Describes current knowledge and factors regarding teen pregnancy in Inuit communities	Portrayed the current determinants of early pregnancy among Inuit youth in the north, in order to better address the topic and aid in creating reproductive health programs
Van Wagner et al. (2012) [17]	No	Nunavik, Quebec, Northern Canada	Data from 1372 labours and 1382 babies from 2002–2007 at the Inuulitsivik birth centre	Northern birthing centres	Maternal-child health outcomes at the Inuulitsivik birth centre	Evaluated the Inuulitsivik birth centre by studying the outcomes of the births, in order to better understand the strengths of maternity services in northern communities

### Theme 1: barriers to reproductive health interventions in the north

Four papers in this review included a general mention of reproductive health interventions that are relevant to Inuit youth and highlighted common barriers to their success in northern remote contexts. Many northern Inuit communities have small populations, where members of the community tend to know each other, for example. Youth reported feeling worried about confidentiality because of this, which was considered a barrier to their reproductive health [12]. Leston and colleagues [12] note that increasing youth’s comfort related to reproductive health topics and issues, as an intervention, is necessary to improve Inuit youth reproductive health overall [12]. In addition, Inuit youth reported a general lack of communication or education regarding sexual and reproductive health. They specifically reported having many unanswered questions about STIs and HIV/AIDS, but felt that no one in their communities were comfortable talking about the subject [12]. Youth requested more communication about reproductive health and specifically wanted easier access to condoms. Educational interventions surrounding reproductive health may be essential in reducing disparities and promoting safe sexual practices [13].

The social determinants of health also impact the reproductive health of northern Inuit youth [14]. Youth in these settings having low income, low levels of education, inadequate housing, living in a single-parent household, low parental education levels, or a history of teenage pregnancy in the family have greater odds of teen pregnancy themselves [3]. Some northern communities in Canada also have higher rates of drug and alcohol use compared to the national rates [12]. This is critical to reproductive health interventions as drug and alcohol use can negatively affect sexual negotiation skills, sexual inhibitions and sexual risk taking, which could lead to unsafe sexual practices, higher STI rates and higher rates of unwanted pregnancies [12, 13]. These potential contextual aspects have implications for the design and implementation of reproductive health interventions for youth in the north.

### Theme 2: northern midwifery

Three articles in this review focused on midwifery and traditional birthing in northern regions of North America. These articles expanded on the need for Inuit midwives as a reproductive health intervention that could be brought back to the north to reclaim traditional birthing. Prior to the 1950s in Canada, Inuit women would give birth in their homes assisted by midwives or other females [6]. Traditional Inuit birthing was known to be a celebration and included ceremonies, gatherings and community traditions [6]. After the 1950s, due to colonial influences and advancements in medical obstetrics, the belief grew among some that midwifery practices were unsafe [6, 15]. Some regions of Canada began preventing midwives from practicing and most funding was cut [6]. Due to the lack of modern obstetrical facilities in regions of the north, most northern women who are expecting a child, including adolescent mothers, are currently transferred by air to urban facilities that have advanced services, at 36 weeks’ gestation [6]. In 1998, a health centre in Nunavik reported that 91% of mothers were transferred to an urban centre to give birth [6]. Currently in Nunavut, that number has lowered to 64.5% of expectant mothers, but is still very high [6]. The outlawing of midwives in the north coincided with the cultural genocide associated with residential schools. These events ultimately contributed to a loss of knowledge on traditional Inuit birthing. This phenomenon has been referred to as “lost births” and this issue remains contentious and unresolved [6].

Couchie and Sanderson [4] highlighted the need to examine and improve birthing services and promote midwifery-driven primary healthcare in the north. The goal of reintroducing midwifery in the north is to restore the significance of birthing traditions without losing the safety of modern obstetrical care. Midwifery requires a 4-year baccalaureate university degree. In Canada, this can be completed at one of seven universities. Once a student has completed this degree, they are certified by the Canadian Association of Midwives and must apply for registration with their respective province or territory. Cardinal [6] notes that Canada also offers eight community-based midwifery programs for individuals who want to learn and practice in their northern communities. These community programs are offered in Nunavut, northern Ontario and northern Quebec. Midwives certified through these programs are not recognized by the Canadian Association of Midwives, but they are able to safely practice in their community as maternity care workers and can gain a diploma in midwifery [6, 15]. These programs have support from the government and from local organizations such as the National Aboriginal Council of Midwives [6]. The current discussion of northern midwifery does not focus specifically on adolescent reproductive health, but it has general relevance to the youth population.

### Theme 3: northern birthing centres

Three articles in this review focused on the evaluation of birthing centres in northern Canada. The Nunavut government has a goal to create more birthing centres in the territory, so the established birthing centres are evaluated in order to identify strengths and weaknesses, to promote the success of future birthing centres [16]. Evaluations of birthing centres in northern regions of North America have largely been Inuit-led with the aim of improving the understanding of the quality and functional scope of maternity services in specific remote communities [17]. Chamberlain and colleagues [1] conducted an evaluation comparing a community with a birthing centre (Community A) to a community with no birthing centre (Community B) [1]. The birthing centre in Community A was staffed with two midwives, an occasional general practitioner, and an Aero-Medical Evacuation Air Service in the case of an emergency. Comparatively, all expectant mothers in Community B were required to transfer to an urban centre to give birth [1]. The results of this study showed that Community A mothers felt less stress during birth due to having family members present and having midwives who could speak their native language. These women were able to participate in making decisions about their health and felt supported psychosocially. Mothers in Community B however, felt higher amounts of stress and isolation giving birth alone in a foreign location, and felt that decisions were made by the health professionals alone with little or no collaboration [1]. The financial implications of these two different approaches were not expended on by these authors, but they would be significant.

Douglas [16] conducted an evaluation of the Rankin Inlet birthing centre, in order to learn from its experiences to potentially inform other new birthing centres in the north [16]. The Rankin Inlet birthing centre is significant as many low risk pregnant women in the Canadian north are transferred to this centre instead of to a southern urban hospital. Between the years 1993–2005, 238 out of 506 births in the Rankin Inlet region took place at this birthing centre [16]. The results of this evaluation showed that the birthing centre has gained political support from the Canadian government and many necessary stakeholders, but seems to lack full support from the community. Rankin Inlet was previously a mining community bringing residents in from many different regions and cultures. This mixture of cultures within the community has an influence on the traditional Inuit culture. The birthing centre has taken on a southern midwifery model [16]. In many ways the centre has become a southern, biomedical institution that does not follow the Inuit birthing traditions [16]. The midwives working at the Rankin Inlet birthing center also rotate through the community from southern Ontario, on fixed terms, so continuity of care is lacking [16]. The authors note that the Inuit women they spoke to seem to value a biomedical birthing model but one that is informed by Inuit cultural and social values. The Inuit women reported valuing a traditional communal birth where the whole community shares in the birth experience, rather than the southern model that revolves around the authority of the expert and biomedical knowledge [16]. Despite these cultural reservations, the article suggests that all low-risk births in the community since the birthing centre’s inception (238 births out of 506 births total) occurred at the centre, demonstrating that the long-term efforts in preventing “lost births” have been successful [16]. Overall, community members reported that community support of the birthing centre is an essential step in ensuring its success, and the success of future birthing centres in other communities [16].

Van Wagner and colleagues [17] conducted an evaluation of the Inuulitsivik Health Centre in Nunavik, Quebec. This birthing centre has long been considered to have successfully returned traditional birthing back to the community. This centre services the general health needs for the communities of Puvirnituq, Inukjuak, and Salluit. In terms of traditional birthing, this health centre has demonstrated best practices in providing traditional midwifery-led birthing care and the education and promotion of local midwives [17]. This health centre has a Perinatal Review Committee which conducts risk screening to determine at-risk pregnancies and also includes a broad practice for midwives. The health centre’s midwives have been considered to have preserved Inuit birthing traditions by passing their midwifery knowledge down through generations. This evaluation showed that the birthing centre has had low rates of birth interventions and overall successful, safe deliveries. They found that 85% of the local births were attended by midwives and only 9% of those births required urgent transfer of the mother and/or infant [17]. Overall, these successful findings demonstrate that Inuit midwifery services can be integrated into northern community birthing centres to safely bring traditional birthing back to the north [17]. Similar to the midwifery theme, the northern-based birthing centre is a reproductive and maternal health intervention that does not focus specifically on adolescent populations, but it is relevant to youth and young mothers in the north.

### Theme 4: fetal fibronectin tests for identifying high-risk pregnancies

Fetal fibronectin tests have recently been utilized to reduce unnecessary travel for expectant mothers in the north [5]. In northern communities, all first-time (nulliparous) mothers are required to evacuate to an urban centre for birth, regardless of whether their community has a birthing centre or not, as all nulliparous births are considered “high-risk”. In 2004, fetal fibronectin tests were introduced as part of an inter-professional pilot project in Iqaluit, Nunavut. In a healthy pregnancy, fetal fibronectin is a glycoprotein in the lining between the mother’s placenta and uterine lining [5]. If the glycoprotein appears in the mother’s vaginal secretions between 25 and 35 weeks’ gestation, it is an indicator that labour could begin prematurely (within the next 7 days) [5]. This test could be an effective indicator on which risk level decisions for nulliparous births could be made. The test results take only 30 min and they are easy to interpret as either a positive or negative [5]. Two years after implementation of these tests, results showed that there were no false negatives and only a 0.4% chance that a mother’s delivery would be premature if her test result came back negative [5]. Between the years 2004–2007, 160 tests were performed, subsequently preventing southern transfers of low-risk nulliparous mothers and saving the Canadian healthcare system hundreds of thousands of dollars [5]. The downfall of these tests is that they are individually costly, with each test costing about $100 [5]. However, as compared to the costs of southern births, this may be nominal. Many first time mothers are younger, especially in northern contexts [3] and so this intervention does have more direct relevance to youth populations.

### Theme 5: prenatal education classes

When expectant mothers of any age in Nunavut travel to urban centres to give birth, they often travel to hospitals and boarding homes in Winnipeg, Manitoba; Churchill, Manitoba; Edmonton, Alberta; Ottawa, Ontario; or Yellowknife, Northwest Territories [18]. There are minimal prenatal education classes in urban centres for these young expectant mothers and Lawford and Giles [18] highlight this gap. Prenatal education improves healthcare workers’ support for the mother and her family, it reduces the mother’s anxiety towards birth, and is a mode for the mother to have questions answered. The Kivalliq Inuit Centre in Winnipeg is a leader in prenatal education classes for Inuit women, and 80% of women from Nunavut travel to this centre to give birth [18]. The Centre has dedicated new space for the classes and has recently provided public health nursing services for education and support. The author highlight that ideally these services should be available in all northern communities, but at the very least could be offered at all boarding homes or hospitals for expectant northern mothers in urban centres in Canada. While programming specifically for adolescent mothers was not specifically mentioned, this may be a context for potential implementation of educational interventions for Inuit youth.

### Theme 6: interventions to improve access to and quality of reproductive health supports

Five articles in this review focused on methods and interventions to improve access to northern reproductive health supports. Many of these articles touched on utilizing long-term community-based participatory research (CBPR) or participatory action research (PAR) as methods to improve sexual and reproductive health in northern Inuit communities. This form of intervention research is developed upon the understanding that positive change is achieved when community members and members of target populations actively participate in, and contribute meaningfully to, intervention and research development [3, 12, 19]. Effective CBPR and PAR approaches have been demonstrated with a focus on reproductive health communication, improving contraception accessibility, providing appropriate training, and involving the correct stakeholders. These could contribute to improved Inuit specific reproductive health programming and outcomes in the north [3, 12, 19] although to date have not focused specifically on youth.

Corosky and Blystad [20] suggest that Inuit youth-specific health programs should be built on the four pillars of Inuit cultural knowledge to improve their cultural competency. These pillars include: Inuuqatigiitsiarniq (being respectful of all people), Unikkaaqatigiiniq (story telling), Pittiarniq (being kind and good), and Iqqaumaqatigiiniq (all things coming into one) [20]. These authors argue that the use of this model could increase trust, promote confidentiality and reduce stigma around reproductive health in Inuit communities [20].

Updating health curriculums in schools and implementing peer educators to teach about teen pregnancy prevention in the community have also proven to be beneficial in improving Inuit reproductive health and this with a youth focus [21]. A curriculum must be non-judgmental, relevant and culturally appropriate. Researchers suggest using inclusive language, LGTBQ2 + content, and promoting positivity in the discussions [21].

Creating a maternal-child health surveillance system for northern populations could also be an effective method for researchers to gather relevant information on reproductive health and aid in reducing health disparities in the north [22]. While not specific to youth per say, the system could include reproductive health information such as maternal nutrition, pregnancy exposures, food security, birth outcomes, and congenital anomalies that could indirectly help in reproductive health promotion for all northern mothers [22]. A participatory approach would allow community members and researchers to communicate their own perspectives about areas of need with respect to reproductive and maternal health [22].

## Discussion

The objective of this scoping review was to provide a summary of literature since the year 2000 that has focused on reproductive health interventions or programs for Inuit adolescents in northern remote communities of North America. Altogether, 17 articles were included. These covered primarily maternal health aspects of reproductive health and the majority were inclusive of youth but adolescents were not the focus of the interventions discussed. The articles were situated in or pertained to Inuit communities in northern regions of Canada and Alaska and the research varied in study design.

Five of the 17 included articles focused on how travelling to give birth can have many negative psychological, social and financial effects on expectant mothers, including adolescents, and their families. Expectant mothers may be expected to live alone for up to three weeks before giving birth in a location with no support system, non-traditional foods, non-native languages, and different birthing traditions and cultures than they are familiar with [6, 23]. Travelling may cause unnecessary financial burdens for a family as well, as currently, any additional people who travel with the mother are required to pay their own airfare and other travel-related expenses [6]. Inuit birthing has departed in many ways from cultural practices in the past decades [24].

Cardinal [6] and James and colleagues [15] noted that prenatal education, breastfeeding support and intentional programs to promote emotional support are rarely offered for Inuit women in urban hospitals. As a result, Inuit mothers may be less informed and uncomfortable asking for assistance. Adolescent mothers may be even more in need of these kinds of education and support given their age and relative lack of life experience and should be a priority in maternal healthcare [3, 24]. These articles suggest that to improve Inuit maternal reproductive health and reduce mothers’ sense of isolation, women should be involved in making decisions about their care and care should be culturally appropriate. Promoting northern community-based midwifery has been suggested as an effective and culturally acceptable method to promote the reproductive health of Inuit women, including teen mothers [6, 15, 25].

Three articles included in this review stated that northern birthing centres staffed with midwives may also be a method to safely and effectively return traditional birthing to the north and aid in improving maternal and reproductive health and reducing health inequities. These articles argued that birthing centres are a safe option for low-risk births in the north and allow the family to experience a culturally traditional birth on home soil [6]. However, there are critiques of the birthing centre model as these centres lack advanced emergency obstetrical care. Emergency protocols and an adapted Medevac system have to be implemented in these communities in cases of an obstetrical emergency [4].

In order to improve Inuit reproductive health from the community-level, multiple authors emphasized an emphasis on improving access to reproductive education and providing youth with effective resources and support in the community. These could include prenatal education classes for young expectant mothers and reproductive health education in schools for students. Prenatal education is fundamental for young mothers and their partners to learn about physiological and psychological changes before, during, and after pregnancy, as well as topics such as breastfeeding and how to promote the health of their infant [18]. Sexual and reproductive health topics are not always areas that members of communities are comfortable talking about [20]. Reducing stigma around these topics could increase youths’ level of comfort when discussing reproductive issues and accessing services. There is in fact a central place for personal, social, health and relationship education for girls and boys in schools. Corosky and Blystad [20] emphasize that young people in the north want to learn more about their health, and reproductive health in particular, and it is the job of northern researchers and public health practitioners to deliver accessible and effective community-based participatory action interventions and school-based programs. Education, community engagement, youth empowerment and self-advocacy is essential for community wellness regarding reproductive health, including that of youth [26, 27].

### Future research recommendations

Areas for future research were identified by several authors. It was recommended that university programs for educating midwives should be offered in rural regions of the north, in addition to the current urban university locations [6]. These authors postulated that a rural, community-based northern program would be most successful if it worked in collaboration with a university with an existing program. Training programs would also require adequate funding, and may benefit from online technology and e-learning.

Luo and colleagues [2] and Moisan and colleagues [3] suggested creating intervention programs that specifically target youth and/or young single mothers. This was a distinct gap in the existing literature. These authors suggested that the most important areas of reproductive health research to focus on were breastfeeding, reducing alcohol use by youth and expectant mothers, drug and smoking use during pregnancy, improving access to health services and contraceptives, and delaying the onset of sexual activity [2, 3]. Additionally, investments should be made in improving the social determinants of reproductive health through enhanced equity, socioeconomic status and housing conditions in northern communities [2].

Finally, Jessen et al. [19] suggested that the North American research community should increase the amount of scientific articles on northern Inuit reproductive health in order to raise awareness about and make a positive impact on reproductive health outcomes overall and for youth specifically. Research needs to have an emphasis on health interventions and programs, improving access to care, addressing social, psychological, and cultural factors, epidemiology, and health indicators. Without quality research and evaluation, providing well-tailored and effective interventions for young people in northern Inuit communities will remain a challenge [19].

### Limitations

It is possible that relevant articles meeting review criteria were missed if documents were not indexed in the academic databases accessed for this review, or if they were not available through Internet or website searching. Relevant articles published before the year 2000 or those that were not written in English were excluded and may have contained additional information about reproductive health interventions for Inuit adolescents. Although we were guided by northern service providers in the topic of this review and we have northern lived-experience on the study team, we did not have an Inuit reviewer involved after the study design. This would have been ideal particularly to assist with interpretation of the knowledge gaps and intervention details as well as in discussion of the broader implications of the study. Also we would also like to emphasize that although all of the included articles and interventions in this review were relevant to Inuit youth, only a small sample of these had youth as a specific focus.

## Conclusion

The volume of literature over the past two decades specifically pertaining to northern reproductive health issues is low and that which is specific to young people aged 10–19 is even more limited. This is an important knowledge gap. The included articles focused almost exclusively on maternal aspects of reproductive health. It is evident that reproductive health concerns remain in the north. There are barriers to the effective implementation of many interventions and limited access to culturally appropriate resources, programs and services. Mothers, including adolescent mothers, often do not have access to maternal health services in their home communities, and limits to the amount and content of reproductive education and programming overall exist, including for youth. There is a distinct need for continued research and intervention programs to support Inuit youth and young mothers with the goal of improving reproductive and maternal health outcomes in the north. There are gaps in knowledge and practice that should not remain unfilled. Implementing culturally appropriate youth-specific programming, midwifery services, in community birthing centres and community-based participatory intervention research could make the most significant long-term impacts to the reproductive health of northern young people.

## Data Availability

The datasets used and/or analysed during the current study are available from the corresponding author on reasonable request.

## Abbreviations

ERIC: Education Resources Information Center
EBSCO: Elton B. Stephens Company
CINAHL: Cumulative Index of Nursing and Allied Health Literature
STI: Sexually transmitted infection
HIV/AIDS: Human immunodeficiency virus / acquired immunodeficiency syndrome
CBPR: Community-based participatory research
PAR: Participatory action research
LGBTQ2 +: Lesbian, gay, bisexual, transgender, queer, and two-spirit

## References

[1] Chamberlain M, Barclay K, Kariminia A, Moyer A (2001). Aboriginal birth: psychosocial or physiological safety. Birth Issues.

[2] Luo ZC, Senécal S, Simonet F, Guimond É, Penney C, Wilkins R (2010). Birth outcomes in the Inuit-inhabited areas of Canada. CMAJ.

[3] Moisan C, Baril C, Muckle G, Belanger RE (2016). Teen pregnancy in Inuit communities–gaps still needed to be filled. Int J Circumpolar Health.

[4] Couchie C, Sanderson S (2007). A report on best practices for returning birth to rural and remote Aboriginal communities. J Obstet Gynaecol Can.

[5] Cavanaugh S. Nursing in Nunavut Part 2. In: Canadian nurse*.* June 1 2009. https://canadian-nurse.com/en/articles/issues/2009/june-2009/nursing-in-nunavut. Accessed 30 May 2020. 19580147

[6] Cardinal MC (2018). “Lost births”, service delivery, and human resources to health. Int J Health Governance.

[7] Reproductive Health. In: World Health Organization-Western Pacific Region. 2020. https://www.who.int/westernpacific/health-topics/reproductive-health#:~:text=Reproductive%20health%20is%20a%20state,to%20its%20functions%20and%20processes. Accessed 20 Jun 2020.

[8] Levac D, Colquhoun H, O'Brien KK (2010). Scoping studies: advancing the methodology. Implement Sci.

[9] Salam RA, Faqqah A, Sajjad N, Lassi ZS, Das JK, Kaufman M, Bhutta ZA (2016). Improving adolescent sexual and reproductive health: a systematic review of potential interventions. J Adolesc Health.

[10] Tricco AC, Lillie E, Zarin W (2018). PRISMA extension for scoping reviews (PRISMA-ScR): checklist and explanation. Ann Intern Med.

[11] Covidence systematic review software, Veritas Health Innovation, Melbourne, Australia. www.covidence.org.

[12] Leston JD, Jessen CM, Simons BC (2012). Alaska Native and rural youths' views of sexual health: A focus group project on sexually transmitted diseases, HIV/AIDS, and unplanned pregnancy. Am Indian Alaska Native Mental Health Res J Natl Center.

[13] Logie CH, Lys CL, Fujioka J, MacNeill N, Mackay K, Yasseen AS (2019). Sexual practices and condom use among a sample of Northern and Indigenous adolescents in Northern Canada: cross-sectional survey results. BMJ Sex Reprod Health.

[14] Logie CH, Lys CL, Okumu M, Fujioka J (2019). Exploring factors associated with condom use self-efficacy and condom use among Northern and Indigenous adolescent peer leaders in northern Canada. Vulnerable Child Youth Stud.

[15] James S, O'Brien B, Bourret K, Kango N, Gafvels K, Paradis-Pastori J (2010). Meeting the needs of Nunavut families: a community-based midwifery education program. Rural Remote Health.

[16] Douglas VK (2011). The Rankin Inlet birthing centre: community midwifery in the Inuit context. Int J Circumpolar Health.

[17] Van Wagner V, Osepchook C, Harney E, Crosbie C, Tulugak M (2012). Remote midwifery in Nunavik, Quebec, Canada: outcomes of perinatal care for the Inuulitsivik health centre, 2000–2007. Birth.

[18] Lawford KM, Giles AR (2016). Kivalliq Inuit Centre boarding home and the provision of prenatal education. Int J Circumpolar Health.

[19] Jessen C, Leston J, Simons B, Rink E (2016). What is missing? Addressing the complex issues surrounding sexual and reproductive health in the circumpolar north. Int J Circumpolar Health..

[20] Corosky GJ, Blystad A (2016). Staying healthy “under the sheets”: Inuit youth experiences of access to sexual and reproductive health and rights in Arviat, Nunavut, Canada. Int J Circumpolar Health.

[21] Hohman-Billmeier K, Nye M, Martin S (2016). Conducting rigorous research with subgroups of at-risk youth: lessons learned from a teen pregnancy prevention project in Alaska. Int J Circumpolar Health.

[22] Lauson S, McIntosh S, Obed N, Healey G, Asuri S, Osborne G, Arbour L (2011). The development of a comprehensive maternal–child health information system for Nunavut-Nutaqqavut (Our Children). Int J Circumpolar Health.

[23] Kaufert PA, O'Neil JD (1990). Cooptation and control: the reconstruction of Inuit birth. Med Anthropol Q.

[24] Archibald L. Teenage pregnancy in Inuit communities: Issues and perspectives. In: Pauktuutit Inuit Women’s Association. 2004. https://www.pauktuutit.ca/wp-content/uploads/TeenPregnancy_e.pdf. Accessed 11 Augt 2020.

[25] Epoo B, Stonier J, Van Wagner V, Harney E (2012). Learning midwifery in Nunavik: community-based education for Inuit midwives. Pimatisiwin.

[26] Jeffery B, Abonyl S, Labonte R, Duncan K (2006). Engaging numbers: developing health indicators that matter for First Nations and Inuit people. Int J Indigenous Health.

[27] Steenbeek A (2004). Empowering health promotion: a holistic approach in preventing sexually transmitted infections among First Nations and Inuit adolescents in Canada. J Holist Nurs.

